# Risk of falling in patients with a recent fracture

**DOI:** 10.1186/1471-2474-8-55

**Published:** 2007-06-28

**Authors:** Svenhjalmar van Helden, Caroline E Wyers, Pieter C Dagnelie, Martien C van Dongen, Gittie Willems, Peter RG Brink, Piet P Geusens

**Affiliations:** 1 Department of General Surgery/traumatology, University Hospital Maastricht, Maastricht, The Netherlands; 2 Department of Epidemiology, Maastricht University, Maastricht, The Netherlands; 3 Department of Internal Medicine, University Hospital Maastricht, Maastricht, The Netherlands; 4 Biomedical Research Institute, Hasselt University, Diepenbeek, Belgium

## Abstract

**Background:**

Patients with a history of a fracture have an increased risk for future fractures, even in short term. The aim of this study was to assess the number of patients with falls and to identify fall risk factors that predict the risk of falling in the first three months after a clinical fracture.

**Methods:**

Prospective observational study with 3 months of follow-up in a large European academic and regional hospital. In 277 consenting women and men aged ≥ 50 years and with no dementia and not receiving treatment for osteoporosis who presented to hospital with a clinical fracture, fall risk factors were assessed according to the guidelines on fall prevention in the Netherlands. Follow-up information on falls and fractures was collected by monthly telephone interview. Incidence of falls and odds ratio's (OR, with 95% confidence intervals) were calculated.

**Results:**

512 consecutive patients with a fracture were regarded for analysis, 87 were not eligible for inclusion and 137 patients were excluded. No follow-up data were available for 11 patients. Therefore full analysis was possible in 277 patients.

A new fall incident was reported by 42 patients (15%), of whom five had a fracture. Of the 42 fallers, 32 had one new fall and 10 had two or more.

Multivariate analysis in the total group with sex, age, ADL difficulties, urine incontinence and polypharmacy showed that sex and ADL were significant fall risk factors. Women had an OR of 3.02 (95% CI 1.13–8.06) and patients with ADL-difficulties had an OR of 2.50 (95% CI 1.27–4.93).

Multivariate analysis in the female group with age, ADL difficulties, polypharmacy and presence of orthostatic hypotension indicated that polypharmacy was the predominant risk factor (OR 2.51; 95% CI: 1.19 – 5.28). The incidence of falls was 35% in women with low ADL score and polypharmacy compared to 15% in women without these risk factors (OR 3.56: CI 1.47 – 8.67).

**Conclusion:**

15% of patients reported a new fall and 5 patients suffered a new fracture within 3 months. Female sex and low ADL score were the major risk factors and, in addition, polypharmacy in women.

## Background

A history of fracture indicates a risk for future fractures. The absolute risk for fractures is highest in the first year after a clinical fracture (fracture with symptoms urging the patient to look for medical attention) [1]. Therefore, the prevention of new fractures should be a part of post-fracture treatment. Fall prevention interventions have been shown not only to reduce certain risk factors for falling, but also have successfully reduced falls [2]. No fall prevention intervention study so far has been large enough to determine whether reducing falls will also reduce the number of fractures.

One third of the people aged 65 years and above fall every year and in one to five percent the fall results in a fracture [3-8]. The prevalence of falls increases with age, and more in women than in men. It is expected that the number of persons who fall and the number of fractures will increase due to the sharp rising in the ageing population. Hip fractures, vertebral fractures and wrist fractures are the most common fractures, but most of the other fractures after the age of 50 are also associated with osteoporosis and falls [8-10]. Fractures after menopause and in elderly can have severe consequences in terms of mortality and morbidity, including admission to hospital or to a nursing home and a decrease in quality of life [6,7,9,11].

Previous research identified the following fall risk factors: age of 65 years and above, female sex, mobility problems, previous falls, muscle weakness, visual impairment, disturbances of the equilibrium, low bone mineral density, multi-medication use such as sedatives, previous fractures, low grip strength, low physical activity, impaired activities of daily living, depression, cognitive impairment, use of assistive devices, urinary incontinence, Parkinson's disease, fear of falling, living in a nursing home, diabetes mellitus, high blood pressure, orthostatic hypotension and others [5,12,10-23]. Certain fall risk factors are modifiable to prevent falls, including visual impairment, physical activity, mobility, and muscle strength [13]. The fall risk factors examined in this study are used as predictors of new falls after a recent clinical fracture. To our knowledge there are no previous studies where fall risk factors were analysed in a cohort of fracture patients.

The aim of this study was to assess the number of patients with falls within 3 months after a recent clinical fracture, and to identify fall risk factors that predict the risk of falling within 3 months after a recent clinical fracture.

## Methods

### Study design and participants

In September 2004, based on the guideline of the Dutch Institute for Health Care Improvement (CBO) on osteoporosis and the guideline on fall prevention, a large European hospital initiated a fracture and osteoporosis outpatient clinic in which fracture patients aged 50 years and above are investigated for osteoporosis [8,10]. The aim of the outpatient clinic is to improve the care of patients with a clinical fracture, to diagnose osteoporosis, and to determine fall risk in fracture patients. Patients who present with a fracture either at the emergency department, at the outpatients clinic or who are hospitalized because of a facture were invited to the fracture and osteoporosis outpatient clinic.

For the present study, all patients with a clinical fracture that visited the hospital for fracture treatment between April and September 2005 were invited to participate. During the first consultation, the nurse specialized in osteoporosis and fall risk assessment informed and invited every patient individually. If the patient agreed to participate in the study, an informed consent form was signed and handed over to the osteoporosis nurse. The second consultation was combined with a dual-energy x-ray absorptiometry (DXA) measurement. During the second visit, patients were informed about the results of the DXA scan and anamnesis for fracture and fall risk assessment were performed. Patients were included if they were willing to undergo 1) a fall risk assessment and 2) a bone mineral density (BMD) measurement by DXA. Patients were excluded for the following reasons: not completing one of both assessments (fall risk and DXA), deceased, established dementia written down in medical history, living in another region, having a pathological (non – osteoporotic) fracture, no informed consent or no show on DXA appointment. Patients already receiving adequate treatment for osteoporosis were not invited to attend the fracture and osteoporosis clinic where the recruitment for the study occurred. The study was approved by the medical ethical committee of the hospital.

### Follow-up and outcome assessment

Follow-up information on falls and fractures was collected by telephone interview, performed monthly for 3 months after the fracture. This three-month period was chosen to evaluate fall risk in the recovery period of the fracture. The osteoporosis nurse asked whether the patient had a fall, and if so, the number and timing of falls and whether the fall resulted in a fracture.

The primary outcome of this study is the incidence of patients who had a fall within the three months of follow-up. Falls were defined as unintentional events which result in a person coming to rest on the floor or a lower level [8,9,22,24]. People who had fallen were classified as a faller or a recurrent faller. A faller was defined as someone who had fallen at least once within 3 months of follow-up, and a recurrent faller as someone who had fallen twice or more within 3 months of follow-up.

### Measurements of risk factors

Fall risk was assessed by measuring balance, mobility, lower limb muscle strength, handgrip strength, cognitive status, activities of daily living, visual impairment and general measurement such as blood pressure. These risk factors were chosen based on their description in the Dutch guideline for prevention of falls in the elderly [8].

Balance was evaluated by the Four-Test Balance Scale, in which the patient was asked to perform feet together stand, semi-tandem stand, tandem stand and one leg stand [25]. If the patient was not able to hold at least one of these positions for 10 seconds, this counted as one fall risk.

Mobility was assessed by the Timed Get Up and Go Test, in which the patient is asked to rise from a chair, walk 3 meters, turn, walk back and sit down in the chair. If the patient was not able to perform the test within 12 seconds this was regarded as a reduced mobility and increased fall risk [26,27].

Lower limb muscle strength was measured by the Chair Stand Test. In this test, the patient was asked to rise up and sit down from a chair as quickly as possible five times, while not using their arms if possible. If the patient was not able to complete the test within two minutes this was regarded as a fall risk [28].

Handgrip strength was measured by the Jamar dynamometer (Jamar, Irvington, NY). The patient was asked to squeeze two times on a handgrip strength indicator with both hands separately. For each hand the maximum score (in kg) was added up. If people squeezed with only one hand, the score of the other hand was replaced by the mean of the group that squeezed with both hands taking sex and dominant hand into account. For women, the cut-off point was < 30 kg, for men the cut-off point was < 50 kg [29].

The Abbreviated Mental Test, a questionnaire to test the cognitive status, was used to assess if patients were cognitive impaired. The cut-off point of this test is 8 (range: 0–10), with a score less than 8 suggesting abnormal cognitive functioning [30,31].

The Groningen Activity Restriction Scale (GARS) was used to test disability in activities of daily living (ADL) [32]. Patients were asked to answer the questions regarding their abilities just before the fracture. The patient was considered as having a fall risk if he or she had difficulties with a least two out of the three following questions of GARS: a) Can you, fully independently, go up and down the stairs; b) Can you, fully independently, walk outdoors (if necessary with a cane); and c) Can you, fully independently, take care of your feet and toenails? Visual impairment was measured by the Snellen eye chart. Patients viewed the eye chart at a distance of 3 meters. If the visual acuity was less than 0.4, the patient was regarded as visually impaired and having a fall risk [33,34].

Blood pressure was measured to determine if orthostatic hypotension was present [35]. It was measured both in lying and standing position (after one minute). Further, patients were asked for previous falls in the past 12 months, the ability to keep their balance, problems with walking, difficulties with rising from a chair, difficulties with dressing and undressing, the use of psychofarmaca, polypharmacy (taking 5 or more pills per day), osteoarthritis (patients were asked for previous medical attention for arthrosis of especially the joint in the lower limb), urinary incontinence (patient was asked for involuntary loss of urine), difficulties with reading the newspaper, and depression.

### Statistical analysis

Statistical analyses were performed using SPSS for Windows version 12.0.1. Individuals without falls were compared with those with falls. First, univariate logistic regression models were fitted with all individual fall risk factors for the total group and women. If a risk factor had an Odds Ratio (OR) ≥ 2.0 it was retained for subsequent multivariate analysis.

The logistic regression analysis was fitted by the "Forward Likelihood Ratio" method. To identify the relationship between the fall risk factors and falling, OR's and 95% confidence intervals were calculated. Interaction was tested for all significant variables resulting from the univariate analysis.

## Results

**Figure 1:**
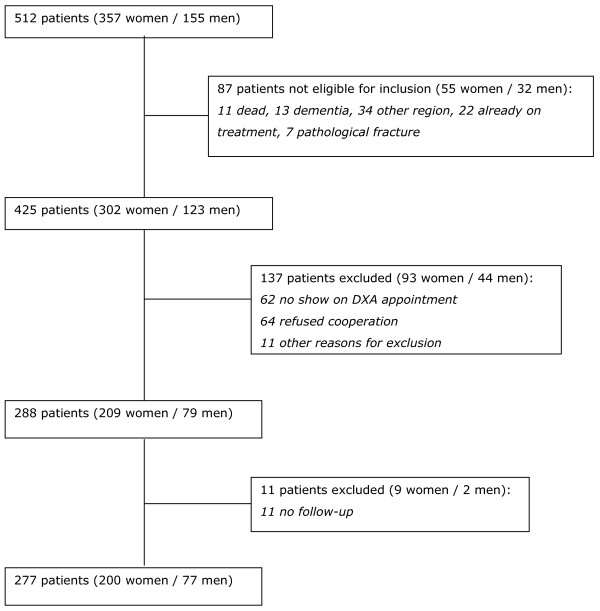
Flowchart with inclusion and exclusion of study participants.

During an inclusion period of 6 months, 512 patients over the age of 50 with a clinical fracture visited the emergency department or the outpatient clinic (Figure 1). Of those patients, 87 were not eligible for inclusion. Of the resulting 425 patients who where eligible for inclusion, 137 patients were excluded for various reasons. No follow-up data were available for 11 patients. As a result, 277 patients were included with a total of 286 clinical fractures at baseline (inclusion rate 65.2% of all eligible patients).

A fall from maximum standing height as the cause of the fracture was reported by 246 patients (88.8%).

In this study the majority of patients were women (72.2%).

A fall within 3 months after a recent clinical fracture was reported by 42 of the patients (15.2%); the number of women with a fall was higher than the number of men (respectively 37 [18.5%] and 5 [6.5%]; p = 0.013). Of the 42 fallers, 10 were recurrent fallers. The fall rate was 1.5 falls/patient year. In 5 patients a fall resulted in a fracture. Mean age of female fallers was 69.9 years (range: 51–86), and 66.6 years in male fallers (range: 51–78) (p = 0.069). All fallers reported that their baseline clinical fracture was the result of a fall from maximum standing height (Table 1). One in two fallers had difficulties with ADL before the clinical fracture, compared with one in four of the patients who had no falls during the follow-up (p = 0.003). The other characteristics were not significantly different between fallers and non-fallers (Table 1). At least one fall risk factor was present in 84% of the patients, 76 (27%) had one fall risk factors, 62 (22%) had two, 47 (17%) three, and 49 (18%) had four or more fall risk factors (table 2).

**Table 1:** Characteristics of Study Population

Characteristic	**Total group (n = 277)**		**Fallers (n = 42)**		**Non fallers (n = 235)**		**p-value**
		
	%	n	%	n	%	n	
Sex (female)	72	200	88	37	69	163	0.013*
**Age, mean (range) years**	67.1 (50–91) ^†^		69.9 (51–86)^†^		66.6 (50–91)^†^		0.069
**Living at home**	93	254	91	38	94	216	0.478
**Low energy trauma**	89	246	100	42	87	204	0.012*
**Bone Mineral Density **^§^							
Normal (Tscore > -1)	24	61	19	7	25	54	0.438
Osteopenia (Tscore ≤ -1 and > -2.5)	47	119	47	17	47	102	0.945
Osteoporosis (Tscore ≤ -2.5)	29	72	33	12	28	60	0.323
**Weight, mean (range) kg**	71.8 (40–120)^†^		71.9 (45–115)^†^		71.8 (40–120)^†^		0.945
**Fracture type**							
Upper extremity	43	119	36	15	44	104	0.430
Lower extremity	48	134	57	24	47	110	0.305
Trunk	5	15	5	2	6	13	0.922
Multiple fractures	3	9	2	1	3	8	0.899
**Diabetes**	17	47	24	10	16	37	0.200
Orthostatic hypotension **	12	33	19	8	11	25	0.235
**Medication**							
Antihypertensive	32	89	41	17	31	72	0.337
Benzodiazepines	9	26	14	6	9	20	0.473
Antidepressive	9	26	12	5	9	21	0.750
Anti-rheumatics	5	13	7	3	4	10	0.649
**Fall risk factors**							
Mobility (TGUGT)^‡^	59	164	71	30	57	134	0.080
≥2 falls in previous year^‡^	30	84	43	18	28	66	0.055
Use of benzodiazepines^‡^	10	28	10	4	10	24	0.891
ADL difficulties^‡^	31	85	50	21	27	64	0.003*
Osteoarthritis^‡^	34	95	31	13	35	82	0.620
Visual impairment^‡^	18	50	24	10	17	40	0.292
Urinary incontinence^‡^	18	50	29	12	16	38	0.054
Parkinson's disease^‡^	1	2	0	0	1	2	0.548

* significant: p < .05

^§ ^results of 252 subjects

** (20 mmHg drop in systolic BP and/or 10 mmHg drop in diastolic BP 1 minute after standing)

^†^Mean (range)

^‡^Fall risk factor according to the Dutch guideline on fall prevention (TGUGT: Timed Get Up and Go Test: cut off at 12 seconds; ADL difficulties: fall risk if he or she had difficulties with a least two out of the three following questions of Gronigen Activity Restriction Scale: a) Can you, fully independently, go up and down the stairs; b) Can you, fully independently, walk outdoors (if necessary with a cane); and c) Can you, fully independently, take care of your feet and toenails?; Visual impaiment: cut off at visual acuity less than 0.4;

**Table 2:** Fall risk factors predicting the risk of falling within 3 months after a fracture: univariate analysis

	Total		Women	
	
	(n = 277)		(n = 200)	
	
	OR*	95% CI	OR*	95% CI
Sex (female)	**3.27**	**1.23–8.66**	-	-
Age (80+ yrs vs. 50–59)	**3.55**	**1.30–9.65**	2.88	0.99–8.40
Mobility (TGUGT)^†‡^	1.88	0.92–3.86	1.75	0.79–3.85
≥2 falls in previous year^†^	1.92	0.98–3.77	1.76	0.86–3.64
Use of benzodiazepines^†^	0.93	0.30–2.82	1.11	0.35–3.55
ADL difficulties^†‡^	**2.67**	**1.37–5.22**	**2.20**	**1.07–4.56**
Arthrosis^†^	0.84	0.41–1.70	0.61	0.28–1.31
Visual impairment^†^	1.52	0.69–3.35	1.03	0.42–2.58
Urinary incontinence^†^	2.07	0.98–4.41	1.49	0.67–3.31
Polypharmacy (≥ 5 pills/day)	**2.58**	**1.30–5.10**	**2.51**	**1.19–5.28**
Orthostatic hypotension^‡^	1.98	0.82–4.74	2.22	0.88–5.60
Cognitive impairment^‡^	1.67	0.76–3.69	1.33	0.55–3.21
Handgrip strength^‡^	1.95	0.94–4.05	1.46	0.64–3.31
FTBS feet together^‡^	1.43	0.71–2.89	1.48	0.68–3.21
FTBS semi-tandem stand^‡^	1.55	0.77–3.11	1.61	0.75–3.45
FTBS tandem stand^‡^	1.36	0.70–2.64	1.39	0.68–2.85
FTBS one leg stand^‡^	1.83	0.91–3.70	1.79	0.81–3.95
Chair stand test^‡^	1.66	0.85–3.26	1.77	0.85–3.68

*Values presented are crude OR's, significant OR's are printed in bold.

^†^Fall risk factors according to the Dutch guideline on fall prevention.

‡ Abbreviations and cut off values: Timed Get Up and Go Test (TGUGT) cut off at 12 seconds. Activities of Daily Living (ADL) difficulties: fall risk if he or she had difficulties with a least two out of the three following questions of Groningen Activity Restriction Scale: a) Can you, fully independently, go up and down the stairs; b) Can you, fully independently, walk outdoors (if necessary with a cane); and c) Can you, fully independently, take care of your feet and toenails?

Visual impairment: cut off at visual acuity less than 0.4.

Orthostatic hypotension: 20 mmHg drop in systolic BP and/or 10 mmHg drop in diastolic BP 1 minute after standing.

Cognitive impairment : Abbreviated Mental Test score ≤ 8.

Handgrip strength : For women, the cut-off point was < 30 kg, for men the cut-off point was < 50 kg.

Four Test Balance Scale (FTBS): If the patient was not able to hold at least one of these positions for 10 seconds, this counted as one fall risk.

Chair stand test: If the patient was not able to complete the test within two minutes this was regarded as a fall risk.

Univariate analysis was carried out for 15 fall risk factors (Table 2). Significant fall risk factors for the total population were sex (OR 3.27/women vs. men; 95% CI 1.23–8.66), age (OR 3.55/80 ^+ ^vs. 50–59 years^; ^95% CI 1.30–9.65), ADL-problems vs. no ADL problems (OR 2.67; 95% CI 1.37–5.22), and polypharmacy vs. no polypharmacy (use of ≥ 5 pills per day) (OR 2.58; 95% CI 1.30–5.10). In women, ADL difficulties (OR 2.20; 95% CI 1.07–4.56) and polypharmacy (OR 2.51; 95% CI 1.19–5.28) were significant fall risk factors.

Multivariate analysis in the total group with sex, age, ADL difficulties, urine incontinence and polypharmacy showed that sex and ADL were significant fall risk factors. Women had an OR of 3.02 (95% CI 1.13–8.06) and patients with ADL-difficulties had an OR of 2.50 (95% CI 1.27–4.93).

Multivariate analysis in the female group with age, ADL difficulties, polypharmacy and presence of orthostatic hypotension indicated that polypharmacy was the predominant risk factor (OR 2.51; 95% CI: 1.19 – 5.28). The incidence of falls was 35% in women with polypharmacy and low ADL score. This risk of falls was three times higher than in women without polypharmacy and with normal ADL score. (OR 3.56; 95% CI 1.47–8.67).

## Discussion

In this study of people over 50 presenting with a fracture, who were not already on osteoporosis treatment, who are not demented and who were able to give informed consent, the incidence of patients with a fall within three months after a clinical fracture was 18.5% in women and 6.5% in men. In 11.9% the fall resulted in a new fracture.

The predominant fall risk factors that predicted a new fall were sex and difficulties in ADL in the total group. In line with other studies, women more often experienced a new fall compared to men [3-8]. Nearly one in three women with polypharmacy as a risk factor had a new fall, compared with one in ten women without this risk factor. The sample was too small to detect differences in fracture.

This study has several limitations. The male group was to small for separate analysis.

The information of the main outcome variable, i.e. falls during follow-up, relied on patients recall of falls, so the possibility of incomplete or biased reports of falls cannot be excluded.

The sample may not be completely typical in view of the various exclusion criteria. Only significant risk factors chosen to measure were described, which by definition can not be exhaustive and some of these assessments were not of the kind that could be carried out within routine clinical practice, but only in a research context.

The follow-up period of three months was short but chosen to evaluate fall risk in the time patients were recovering from their fracture.

Based on the results of our study, a health care provider can identify patients with the highest risk of falls after a fracture examining ADL in all patients, and polypharmacy in women.

Future studies should be large enough to include sufficient numbers of men and perform subgroup analysis on different ages. Eventually a very large cohort analysis with fracture risk as endpoint would be most interesting.

## Conclusion

Patients with a recent clinical fracture have a high risk for new falls. In a group of 277 patients, 15% reported a new fall and 5 patients suffered a new fracture within 3 months. Female sex and low ADL score were the major risk factors and, in addition, polypharmacy in women.

## Competing interests

SH is member of an advisory board of Merck Sharp Dohme. SH and PG received an unrestricted grant for research by MSD (not current study).

All other auhors have no conflict of interest

## Authors' contributions

SH coordinated study concept and design, performed acquisition of data together with analysis and interpretation and prepared the final manuscript. CW performed analysis and interpretation of data and took part in manuscript preparation. PD took part in analysis and interpretation of data and preparation of the manuscript. MD performed analysis and interpretation of data together with preparation of the manuscript. GW did acquisition of subjects and data. PB took part in preparation of the final manuscript. PG was involved in study concept and design, analysis and interpretation of data and preparation of the final manuscript. All authors read and approved the final manuscript.

## Pre-publication history

The pre-publication history for this paper can be accessed here:


